# Bolusing frequency and amount impacts glucose control during hybrid closed‐loop

**DOI:** 10.1111/dme.13436

**Published:** 2017-08-19

**Authors:** L. Bally, H. Thabit, Y. Ruan, J. K. Mader, H. Kojzar, S. Dellweg, C. Benesch, S. Hartnell, L. Leelarathna, M. E. Wilinska, M. L. Evans, S. Arnolds, T. R. Pieber, R. Hovorka

**Affiliations:** ^1^ Wellcome Trust–MRC Institute of Metabolic Science University of Cambridge Cambridge UK; ^2^ Department of Diabetes & Endocrinology Cambridge University Hospitals NHS Foundation Trust Cambridge UK; ^3^ Department of Diabetes & Endocrinology Clinical Nutrition and Metabolism, Inselspital Bern University Hospital University of Bern Bern Switzerland; ^4^ Department of Paediatrics University of Cambridge Cambridge UK; ^5^ Department of Internal Medicine Division of Endocrinology & Diabetology Medical University of Graz Graz Austria; ^6^ Profil Institut fuer Stoffwechselforschung GmbH Neuss Germany; ^7^ Central Manchester University Hospitals NHS foundation Trust and University of Manchester Manchester UK

## Abstract

**Aim:**

To compare bolus insulin delivery patterns during closed‐loop home studies in adults with suboptimally [HbA_1c_ 58–86 mmol/mol (7.5%–10%)] and well‐controlled [58 mmol/mol (< 7.5%)] Type 1 diabetes.

**Methods:**

Retrospective analysis of daytime and night‐time insulin delivery during home use of closed‐loop over 4 weeks. Daytime and night‐time controller effort, defined as amount of insulin delivered by closed‐loop relative to usual basal insulin delivery, and daytime bolus effort, defined as total bolus insulin delivery relative to total daytime insulin delivery were compared between both cohorts. Correlation analysis was performed between individual bolus behaviour (bolus effort and frequency) and daytime controller efforts, and proportion of time spent within and below sensor glucose target range.

**Results:**

Individuals with suboptimally controlled Type 1 diabetes had significantly lower bolus effort (*P* = 0.038) and daily bolus frequency (*P* < 0.001) compared with those with well‐controlled diabetes. Controller effort during both daytime (*P* = 0.007) and night‐time (*P* = 0.005) were significantly higher for those with suboptimally controlled Type 1 diabetes. Time when glucose was within the target range (3.9–10.0 mmol/L) during daytime correlated positively with bolus effort (*r* = 0.37, *P* = 0.016) and bolus frequency (*r* = 0.33, *P* = 0.037). Time when glucose was below the target range during daytime was comparable in both groups (*P* = 0.36), and did not correlate significantly with bolus effort (*r* = 0.28, *P* = 0.066) or bolus frequency (*r* = –0.21, *P* = 0.19).

**Conclusion:**

More frequent bolusing and higher proportion of insulin delivered as bolus during hybrid closed‐loop use correlated positively with time glucose was in target range. This emphasises the need for user input and educational support to benefit from this novel therapeutic modality.

## What's new?

- Glucose control during hybrid closed‐loop therapy is linked to bolusing behaviour.
- Closed‐loop users with well‐controlled Type 1 diabetes bolused more frequently without increasing the risk of hypoglycaemia.
- We highlight the importance of user input and education to gain optimal benefit from hybrid closed‐loop.

**What's new?**

- Glucose control during hybrid closed‐loop therapy is linked to bolusing behaviour.
- Closed‐loop users with well‐controlled Type 1 diabetes bolused more frequently without increasing the risk of hypoglycaemia.
- We highlight the importance of user input and education to gain optimal benefit from hybrid closed‐loop.

## Introduction

The need for optimal glycaemic control to avoid diabetes‐related complications is well established 1, 2. Efforts to tighten glycaemic control, however, often result in increased risk of hypoglycaemia 3. Closed‐loop insulin delivery is an emerging diabetes technology that has the potential to address the unmet clinical need for improved glucose control while reducing the burden of hypoglycaemia and self‐care in Type 1 diabetes 4. Closed‐loop use during free‐living unsupervised condition across various patient populations with Type 1 diabetes has been shown to be efficacious 5, 6, 7, 8, and demonstrated greater benefits of closed‐loop during night‐time compared with daytime.

The hybrid closed‐loop approach in these studies employs manual administration of a pre‐meal bolus to mitigate delayed absorption of rapid‐acting insulin analogues. We hypothesised that bolusing behaviour may impact daytime glucose control. The aim of the analysis is to compare insulin delivery patterns from two previous closed‐loop home studies in adults with suboptimally controlled (group 1) 6 and well‐controlled (group 2) Type 1 diabetes 9.

## Methods

We retrospectively analysed daytime (07:00 to 23:00) and night‐time (23:00 to 07:00) insulin delivery in participants who used closed‐loop at least 85% of the time over 4 weeks from two separate closed‐loop home studies (ClinicalTrials.gov registration numbers NCT01961622 6 and NCT02727231 9). The inclusion and exclusion criteria were similar for both study groups, with the exception of HbA_1c_ 58–86 mmol/mol (7.5%–10%) in group 1, and < 58 mmol/mol (< 7.5%) in group 2. The closed‐loop system used in both studies was identical comprising Dana Diabecare R insulin pump (Sooil, Seoul, South Korea), FreeStyle Navigator II continuous glucose monitor (Abbott Diabetes Care, Alameda, CA, USA) and model predictive controller (University of Cambridge, UK) residing on a smartphone (Galaxy S4, Samsung, South Korea) communicating wirelessly with continuous glucose monitoring receiver through a purpose made translator unit (Triteq, Hungerford, UK). Daytime and night‐time controller effort, defined as the amount of insulin delivered by closed‐loop relative to usual basal insulin delivery, and daytime bolus effort, defined as total bolus insulin delivery relative to total daytime insulin delivery were calculated.

Group 1 and group 2 data were contrasted using an independent sample *t*‐test. Correlation analysis was performed between individual bolus behaviour (bolus effort and frequency) and daytime controller efforts, proportion of time spent within (3.9–10.0 mmol/L) and below sensor glucose target range. Outcomes were calculated using GStat software, version 2.2.4 (University of Cambridge, UK) and statistical analyses were performed using SPSS version 23 (IBM Software, Winchester, UK). Data are reported as mean (sd); *P*‐values < 0.05 were considered statistically significant.

## Results

Data from 459 days and 701 nights from group 1 (*N* = 32), and 421 days and 579 nights from group 2 (*N* = 29) were analysed. Baseline characteristics for group 1 and group 2 were: HbA_1c_ 69 (7) mmol/mol [8.4 (0.6)%] vs. 52 (5) mmol/mol [6.9 (0.5)%], *P* < 0.001; female : male 15 : 17 vs. 15 : 14, age 40 (19) vs. 41 (13) years, *P* = 0.81; BMI 25.4 (4.4) vs. 25.1 (3.0) kg/m^2^, *P* = 0.76; duration of diabetes 21 (9) vs. 24 (12) years, *P* = 0.32; duration of pump use 8 (6) vs. 6 (4) years, *P* = 0.20; and pre‐study total daily insulin 0.61 vs. 0.53 U/kg/day, *P* = 0.036.

**Figure 1:**
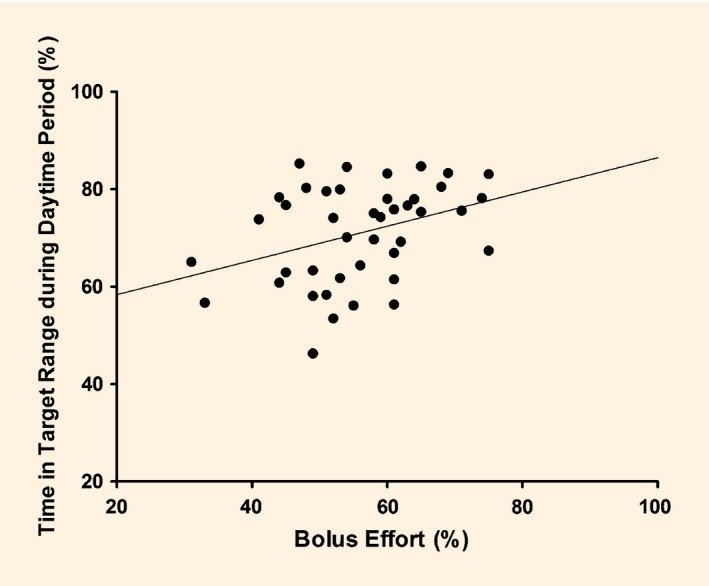
Correlation between % time spent in target range during the daytime period and bolus effort.

Group 1 had a significantly lower bolus effort [53 (8) vs. 59 (11)%, *P* = 0.038] and daily bolus frequency [4.7 (1.1) vs. 6.0 (1.5), *P* < 0.001] compared with group 2 (Table 1). This was accompanied by significantly higher controller efforts during both daytime and night‐time for group 1 compared with group 2 [daytime: 120 (22) vs. 104 (14)%, *P* = 0.007; night‐time: 161 (39) vs. 135 (26)%, *P* = 0.005]. Proportion of time spent in sensor glucose target range during the daytime period correlated positively with bolus effort (*r* = 0.37, *P* = 0.016; Fig. 1) and bolus frequency (*r* = 0.33, *P* = 0.037). Time spent below sensor glucose target range during daytime was comparable in both groups (*P* = 0.36), and did not correlate significantly with bolus effort (*r* = 0.28, *P* = 0.066) or bolus frequency (*r* = –0.21, *P* = 0.19). There was a trend towards an inverse relationship between mean glucose and (i) bolus frequency (*r* = –0.23, P = 0.082) and (ii) bolus effort (*r* = –0.29, *P* = 0.062). For each bolus, there was a trend for an associated reduction in mean glucose of 0.21 mmol/L (*P* = 0.082). Overall and night‐time variability in insulin requirements were comparable between group 1 and group 2.

**Table 1:** Characteristics of insulin delivery and glucose control during closed‐loop insulin delivery in suboptimally controlled (group 1, screening HbA_1c_ > 58 mmol/mol or 7.5%) and well‐controlled (group 2, screening HbA_1c_ < 58 mmol/mol or 7.5%) Type 1 diabetes

	Suboptimally controlled Group 1	Well‐controlled Group 2	*P*‐value
Daytime time in target range, 3.9–10mmol/l (%)	67 (11)	75 (7)	0.008
Daytime time below target range (%)	2.4 (1.0–4.4)	2.8 (2.0–4.8)	0.29
Bolus effort per 24 h period (%)	53 (8)	59 (11)	0.038
Bolus frequency per 24 h (*n*)	4.7 (1.1)	6.0 (1.5)	< 0.001
Controller effort (%)			
Night‐time (23:00 to 07:00)	161 (39)	135 (26)	0.005
Daytime (07:00 to 23:00)	120 (22)	104 (14)	0.007
CV of insulin requirements night‐time (%)	33 (8)	33 (7)	0.76
CV of insulin requirements 24 h period (%)	17 (4)	17 (3)	0.86

Data are calculated from participants who used closed‐loop for at least 85% of the time and are presented as mean (sd) or median (interquartile range). Daytime: *n* = 22 (group 1) and *n* = 21 (group 2); night‐time: *n* = 31 (group 1) and *n* = 27 (group 2); 24‐h period: *n* = 24 (group 1) and *n* = 21 (group 2).

Bolus effort = proportion of total bolus insulin relative to total daily insulin during the daytime from 07:00 to 23:00.

Controller effort = proportion of insulin amount delivered by closed‐loop relative to usual basal insulin amount.

Insulin requirements = proportion of total daily insulin requirements (bolus and closed‐loop delivery) relative to usual total daily dose.

CV, coefficient of variation.

## Discussion

The result of this retrospective analysis shows that hybrid closed‐loop users with well‐controlled Type 1 diabetes have higher bolusing frequency and total amount of self‐administered bolus insulin relative to total daytime insulin, compared with those with suboptimally controlled diabetes. This bolusing behaviour was associated with greater time spent within the glucose target range. However, no relationship was found between greater bolusing efforts and behaviour, with time spent below target glucose range.

Previously published day‐and‐night closed‐loop home studies have shown that the magnitude of glucose outcome improvements during the daytime period was less pronounced compared to the overnight period 6, 10, 11. This is likely due to meal‐time glycaemic excursions and physical activity related changes in insulin sensitivity, and limitations of closed‐loop performance imposed by currently available rapid‐acting insulin pharmacokinetics and the lag in interstitial fluid glucose appearance 12. Meal‐announcements to the closed‐loop control algorithm and meal‐time insulin bolusing, such as that in a hybrid closed‐loop system, may partly mitigate against post‐meal glycaemic excursions.

However, such user‐driven input is dependent on compliance and appropriate self‐management behaviours by the user, to avoid post‐meal hyperglycaemia and/or delayed hypoglycaemia. A recent study evaluating the relationship between behavioural patterns and glucose control (mean glucose, glucose variability and post‐intervention HbA_1c_) during hybrid closed‐loop use in suboptimally controlled Type 1 diabetes highlighted the impact behavioural patterns, such as eating habits, have on glucose control 13. In line with the present study, no significant association was found between bolusing frequency and mean glucose. However, these relationships were not explored in well‐controlled Type 1 diabetes individuals at increased risk of hypoglycaemia [HbA_1c_ <58 mmol/mol (< 7.5%)]. The need to self‐administer insulin boluses for meals may also not match user's initial expectation of an ‘artificial pancreas’, or as an approach to lessen their self‐management burden 14.

Potential alternative approaches include ultrafast‐acting insulin 15, adjuvants such as amylin which delays meals absorption 16, or local warming devices to enhance insulin absorption 17. Prior to adopting these approaches into wider clinical practice, large‐scale studies are still needed to demonstrate their clinical efficacy and user acceptability. The first commercially available closed‐loop system recently approved by the US Food and Drug Administration adopts the hybrid approach 18. As such, hybrid closed‐loop remains at present a pragmatic and well‐studied therapeutic approach for bringing closed‐loop into clinical practice. Owing to the requirement for on‐going user input and interaction with the system, however, there is a continuing need for education and training in this novel technology to help manage user expectations and gain optimal benefit in clinical practice 14.

Intensive insulin therapy and tight glycaemic control are associated with increased hypoglycaemia risk 3. In daily practice, this may be due, in part, to insulin boluses related to meals or corrections, which were over‐estimated or ill‐timed 19. In the present analysis, time spent below target was found to be comparable between two cohorts, despite the relatively higher bolusing frequency in the cohort with lower HbA_1c_. The autonomous modulation of insulin delivery by closed‐loop control algorithm in a glucose‐responsive manner, which also accounts for events having a protracted influence on glycaemia such as manually delivered meal‐time and correction boluses 4, highlights the advantage of closed‐loop use compared to conventional insulin therapy.

The strength of this analysis is the large dataset of hybrid closed‐loop use available during unsupervised free‐living conditions over 4 weeks, from two Type 1 diabetes cohorts with different glycaemic control levels. This allows for characterisation and assessment of bolusing behaviours from these two cohorts, during unbiased real‐world use of hybrid closed‐loop. The hybrid closed‐loop systems used in both studies were similar, thereby enabling the differences between user‐driven behaviours to be analysed independent of the system used. The analysis of bolusing behaviour was limited by the lack of distinction between boluses which were meal‐related, or solely as corrections for elevated glucose levels. Bolus calculator use, carbohydrate‐counting skills and activity levels between the two groups may have differed, thereby possibly confounding the results. The data pertaining to overall bolusing frequency and efforts, however, underline the importance of bolusing behaviour to the benefits and glycaemic outcome of hybrid closed‐loop. Reliable data related to meal sizes and physical activity were not available, and are not reported.

In conclusion, the benefit of hybrid closed‐loop during the daytime period is associated with bolusing behaviour by users. It is of importance to emphasise user input and education, if the benefit from hybrid closed‐loop application is to be optimised in clinical practice.

## Funding sources

Seventh Framework Programme of the European Union (ICT FP7‐ 247138). Additional support for the Artificial Pancreas work by JDRF, National Institute for Health Research Cambridge Biomedical Research Centre, Wellcome Strategic Award (100574/Z/12/Z), EC Horizon 2020 (H2020‐SC1‐731560), NIDDK (DP3DK112176 and 1UC4DK108520‐01), Efficacy and Mechanism Evaluation Programme of National Institute for Health Research (14/23/09) and Helmsley Trust (2016PG‐T1D045 and #2016PG‐T1D046). LB received support from the Swiss National Science Foundation (P1BEP3_165297).

## Competing interests

RH reports having received speaker honoraria from Eli Lilly, Novo Nordisk and Astra Zeneca, serving on advisory panel for Eli Lilly and Novo Nordisk, receiving license fees from BBraun and Medtronic, and having served as a consultant to BBraun. MEW has received license fees from Becton Dickinson and has served as a consultant to Beckton Dickinson. MLE reports having received speaker honoraria from Abbott Diabetes Care, Novo Nordisk and Animas, serving on advisory panels for Novo Nordisk, Abbott Diabetes Care, Medtronic, Roche and Cellnovo, and holding stock options in Cellnovo. SH serves as a consultant for Novo Nordisk and for the ONSET group, and reports having received speaker/training honoraria from Medtronic. RH and MEW report patents and patent applications. JKM reports having received speaker honoraria from Abbott Diabetes Care, AstraZeneca, Eli Lilly & Co, NintaMed, Novo Nordisk, Roche Diabetes Care, Sanofi, Servier, Takeda, and serving on advisory panel for Becton Dickinson, MSD, Sanofi and Boehringer Ingelheim. TRP is an advisory board member of Novo Nordisk A/S, a consultant for Roche, Novo Nordisk A/S, Eli Lilly & Co, Infineon, Carnegie Bank and on speaker's bureau of Novo Nordisk A/S and Astra Zeneca. LL reports having received speaker honoraria from Minimed Medtronic, Animas, Sanofi and Novo Nordisk, and serving on advisory panel for Animas Minimed Medtronic and Novo Nordisk. LB, HT, SD, CB, MH, HK and SA declare that no competing financial interests exist.

### Author contributions

LB, HT and RH had full access to all of the data in the studies and take responsibility for the integrity of the data and the accuracy of the data analysis. LB, HT and RH co‐designed the analysis. RH, MLE, LL, CB, SA, HT and MEW co‐designed the clinical studies. LB, HT, SH, SD, JKM, MH, HK and JP were responsible for screening and enrolment of participants, and arranged informed consent from the participants. LB, HT, SH, SD, JKM, MH and HK provided patient care and/or took samples. LB and HT carried out data analysis. LB, HT and RH wrote the manuscript. All authors critically reviewed the report.
